# Canine oral melanoma with suspected pulmonary metastasis: Combination of immunotherapy and tyrosine kinase inhibitor treatment

**DOI:** 10.17221/90/2023-VETMED

**Published:** 2023-12-26

**Authors:** Kyung-Ryung Kim, Dong-Hyun Kim, Min-Jung Jung, Dong-Min Sihn, Soon-Wuk Jeong, Jung-Hyun Kim

**Affiliations:** ^1^KU Animal Cancer Center, Konkuk University Veterinary Medical Teaching Hospital, Seoul, Republic of Korea; ^2^Department of Veterinary Internal Medicine, College of Veterinary Medicine, Konkuk University, Seoul, Republic of Korea; ^3^Department of Veterinary Surgery, College of Veterinary Medicine, Konkuk University, Seoul, Republic of Korea

**Keywords:** dog, imatinib, malignant melanoma

## Abstract

This case report follows a 10-year-old castrated male Miniature Schnauzer dog presented with a history of incomplete surgical resection of an oral malignant melanoma (amelanotic type) on the right mandible. Melanoma vaccine therapy was administered due to incomplete surgical resection, however, new masses were detected on the contralateral mandible and suspected pulmonary metastasis occurred at 2 weeks and 7 months, respectively, following the first melanoma vaccination. At the time of detecting the pulmonary metastasis, targeted chemotherapy was initiated with the owner’s consent using imatinib (10 mg/kg/day, p.o.), a tyrosine kinase inhibitor (TKI). The patient did not show any significant adverse events related to both anticancer treatments. Three months following the first dose of imatinib, the absence of the suspected pulmonary metastatic nodules on radiography indicated complete remission. In conclusion, this report describes the achievement of clinical remission of suspected pulmonary metastatic oral malignant melanoma and an extension of survival time in a dog given a combination treatment of immunotherapy and tyrosine kinase inhibitor.

Oral melanoma is the most common oral malignancy in dogs. The biological behaviour of this tumour is extremely variable depending on the anatomic location, stage, size, and histologic features. Significant poor prognostic indicators of canine oral melanoma include advanced clinical stage, large tumour size, evidence of metastasis, high mitotic index, nuclear atypia, and the presence of necrosis or inflammation (Bergman 2007). Mucosal melanoma, including oral melanoma, is considered malignant because of its tendency to infiltrate local tissue and metastasize to regional lymph nodes, lungs, and other tissues (Bergman 2007). Treatment options for dogs with melanoma include surgical excision, chemotherapy, radiation therapy, and immunotherapy. However, conventional chemotherapy and radiotherapy as sole treatments have failed to demonstrate significant improvement in survival time in veterinary patients (Proulx et al. 2003; Dank et al. 2014). Although controversial DNA vaccines against tumour antigens have shown better outcomes than conventional anticancer treatment options (Pellin 2022).

Tyrosine kinase inhibitors (TKIs) target protein kinases, which play crucial roles in cell signal transduction, proliferation, differentiation, and other regulatory mechanisms. TKIs are called targeted cancer therapy because they specifically act against the intracellular ATP-binding domains of the membrane-bound receptor tyrosine kinase (Thomas et al. 2012). Receptor tyrosine kinases (TKIs), such as platelet-derived growth factor receptors (PDGFR) are candidates for molecular targeted therapy. TKIs bind to the ATP-binding site of tyrosine kinase proteins, blocking the initiation of downstream signalling cascades in cancer cells. Imatinib is one of these TKIs and has an inhibitory activity against ABL, BCR-ABL, PDGFR, and c-KIT. Therefore, it has benefits as a treatment for numerous tumours associated with overexpression of PDGFR and c-KIT. In human medicine, imatinib is well known as a treatment for chronic myeloid leukaemia, by preventing BCR-ABL protein from signalling oncogenic pathways (Sacha 2014). Imatinib therapy has also been trialled for managing tumours characterised by overexpression of c-KIT in human medicine, including metastatic melanoma and oral mucosal melanoma (Carvajal et al. 2011; Deinlein et al. 2017).

Administration of imatinib to various veterinary oncology patients has been used to treat mast cell tumours, gastrointestinal stromal tumours, squamous cell carcinomas, and fibrosarcoma (Lachowicz et al. 2005; Bonkobara 2015). However, to our knowledge, there has not been a reported use in canine oral melanoma with suspected pulmonary metastasis.

## Case description

This report describes the clinical outcome of a canine case that received a combination of melanoma vaccine and imatinib therapy for an incompletely excised amelanotic melanoma with suspected pulmonary metastasis.

**Figure 1:**
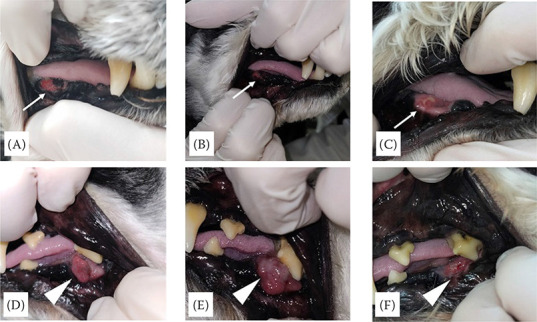
Oral masses on both sides of the mandible Macroscopic lesions of the right mandibular oral mass at 1 month (A), 3 months (B), and 5 months (C) following surgical resection. Macroscopic lesions of a newly detected left mandibular oral mass at 1 month (D), 3 months (E), and 5 months (F) following surgical resection

**Figure 2:**
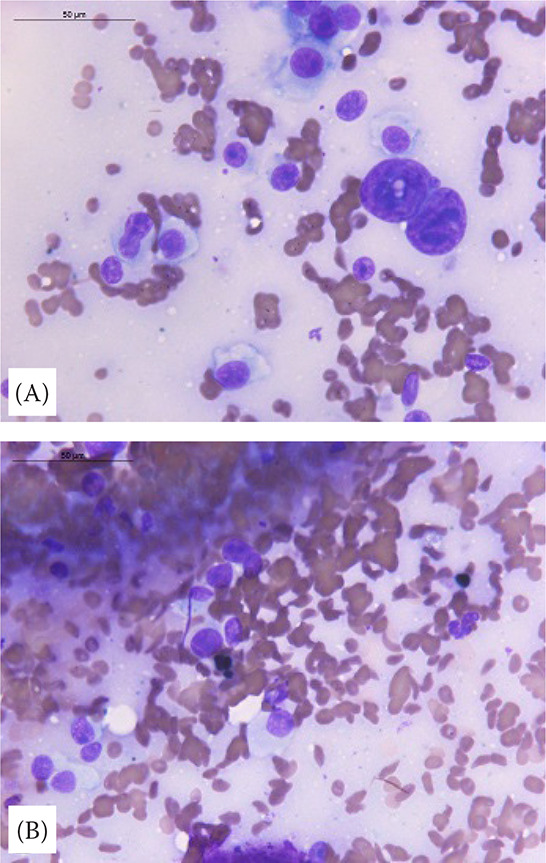
Cytological images of the recurrent oral mass on the right mandible (A), and the newly detected mass on the left mandible. (B) Epithelioid cells were characterised by multiple nucleoli, macronucleus, irregular chromatin, and variable N : C ratio (arrows). In addition, blue-black coloured material appears focally on the background of the sample. Haematoxylin-eosin staining; original magnification **× **400

**Figure 3:**
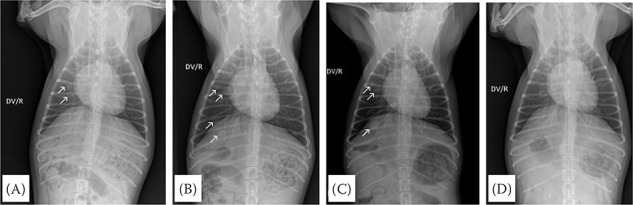
Progression and remission of suspected pulmonary metastatic nodules following imatinib treatment (A) Repeat thoracic radiography at 6 months after recurrence of the oral mass showing 2 identifiable nodules suspected to be pulmonary metastasis (arrows). (B) One month later, 4 nodules were detected (arrows). (C) Two months after the first administration of imatinib, partial remission of the suspected pulmonary metastatic nodules is detected on radiography (arrows). (D) About 3 months after the initiation of chemotherapy, no pulmonary nodules are detected on thoracic radiography

A 10-year-old castrated male Miniature Schnau-zer dog presented with a history of debulking surgery of an oral mass (1.5 × 1.5 cm) on the right side of the caudal mandible 2 weeks prior, which had been diagnosed as an oral malignant amelanotic melanoma. At the time of the patient’s visit, the complete blood count was unremarkable, and the serum biochemical profile showed a mild increase in liver enzyme activity, including aspartate transaminase (AST, 3.54 μkat/l; reference range: 0–0.86 μkat/l) and alanine aminotransferase (ALT, 4.93 μkat/l; reference range 0.17–1.66 μkat/l). Computed tomography performed to evaluate the local invasion and metastatic spread of the head, neck, and thorax showed osteolytic changes to the lateral aspect of the right mandible and a mild enlargement of the right mandibular and medial retropharyngeal lymph nodes, which led to a highly suggestive tumour invasion and regional lymph node involvement. According to the WHO system, the patient was staged as stage III, and a unilateral mandibulectomy (right side) was recommended. However, the owner refused the surgery due to concerns about anaesthesia. Instead, melanoma vaccine (Oncept^®^; Boehringer Ingelheim Animal Health USA Inc., Duluth, GA, USA) therapy was initiated with 4 doses, given 2 weeks apart. When the patient presented for the second vaccination, local progression of the oral mass on the right mandible (0.9 × 0.5 cm) and a newly detected mass (1.4 × 0.9 cm) on the left mandible were observed (Figure 1). Fine needle aspirates of the right and left mandibular oral masses were obtained, and malignant melanoma (amelanotic, epithelioid type) was diagnosed (Figure 2). Although the oral melanoma relapsed, 8 weeks of melanoma vaccination therapy was completed with the owner’s consent, and regular check-ups were continued. No significant adverse events occurred during melanoma vaccine therapy. Five months after that, a monthly routine thoracic radiography revealed the development of suspect pulmonary metastasis with 2 pulmonary masses (11 × 10 mm average size) (Figure 3). The number of pulmonary nodules increased to 4 in a month, however, no relevant clinical signs were observed.

At this time, with the owner’s consent, targeted chemotherapy was initiated using imatinib. Imatinib therapy was started at 5 mg/kg, p.o., q24 h for 14 days. After that, the dose was increased to 7.5 mg/kg, p.o., q24 h for 14 days, then 10 mg/kg, p.o., q24 hours. The patient was monitored weekly for any possible adverse events, including anaemia, neutropenia, and kidney or liver toxicity; and imatinib was well tolerated by the patient. Two months after the initiation of imatinib therapy, a decrease in the average size of the pulmonary nodules was noted, from 15 × 14 mm to 13 × 10 mm, and the number of pulmonary nodules was reduced from 4 to 3. After another month, no pulmonary nodules were detected on thoracic radiography (Figure 3).

**Figure 4:**
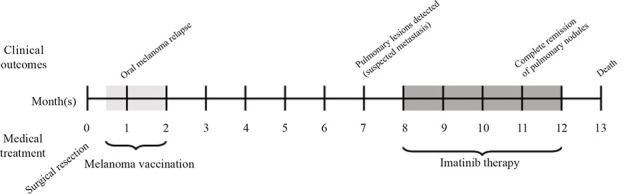
Timeline of medical therapy and clinical outcomes in a dog with oral malignant melanoma Two weeks after incomplete surgical resection of an oral melanoma, melanoma vaccination therapy was initiated. Suspected local recurrence and metastasis were detected during vaccination. Five months after the last vaccine injection, pulmonary nodules were detected on regular radiographic examination. Following this, 4 months of imatinib therapy was initiated by increasing the imatinib titre to 10 mg/kg, q24 hours. During the imatinib therapy, blood and radiographic examination was performed once a week. Three months after administering imatinib, complete remission of the pulmonary nodules was observed on regular radiographic monitoring. The patient died one month after discontinuation of imatinib treatment

Although there was radiographic resolution in the pulmonary lesions, the oral lesions progressed during imatinib therapy; and the patient developed severe neoplastic cachexia. Imatinib therapy was discontinued, and only palliative care was done due to the owner’s request. The patient died at home, 1 month after discontinuation of imatinib and 13 months after diagnosis (Figure 4). Unfortunately, an autopsy was not performed due to the owner’s refusal.

## DISCUSSION

Although oral malignant melanoma is a common disease in dogs, no standard treatment in veterinary patients has been established due to the highly metastatic and recurrent nature of the tumour (Verganti et al. 2017). A study showed that conventional chemotherapy using carboplatin in dogs with oral melanoma is not effective at preventing metastasis or prolonging survival (Verganti et al. 2017). To overcome the limitations of conventional treatment for canine oral melanoma, the canine melanoma vaccination has been studied in veterinary medicine. The canine melanoma vaccination is a xenogeneic vaccination that targets tyrosinase, a rate-limiting enzyme that is overexpressed in malignant cells and is essential for the production of melanin. However, this newly developed immunotherapy showed variable efficacies in altering the inherent biological behaviour of canine oral malignant melanoma according to some previous studies (Treggiari et al. 2016; Verganti et al. 2017).

A combination of immunotherapy and TKI has been tried on numerous types of tumours in human medicine, including mucosal melanoma, lung squamous cell carcinoma, renal cell carcinoma, non-small cell lung cancer, and breast cancer (Wilmott et al. 2012; Aris and Barrio 2015; Ahn and Ursini-Siegel 2021). Moreover, a previous study conducted on a dog with lingual melanoma showed a marked extension of survival time after applying a combination treatment of melanoma vaccine and tyrosine kinase inhibitors (Berry et al. 2022). The combination strategy of TKI and immunotherapy is considered more effective due to various mechanisms, including promoting cell apoptosis, enhancing drug sensitivity, and inhibiting the development of resistance (Yang and Tam 2018).

Recently, TKI has been sporadically administered to various malignant cancers (Smedley et al. 2021). TKI is highly selective against specific molecular targets known to be the cause of the establishment and maintenance of tumour malignancy. Since each TKI targets specific cellular molecules, it is important to choose the appropriate TKI for each patient. Canine oral malignant melanomas tend to have a poor prognosis despite surgical resection, and several studies have identified overexpression and mutation of receptor tyrosine kinases as the potential cause (Chu et al. 2013; Gramer et al. 2016). Imatinib is a multi-kinase inhibitor including ABL, c-KIT, and PDGFR. Imatinib was administered as a targeted chemotherapy drug for the present patient based on a previous study that confirmed PDGFR expression in canine oral malignant melanoma tissue samples (Iussich et al. 2017). In that study, PDGFR-α and β expression rates were 54.2% and 47.9%, respectively. Among the 48 cases analysed, 37.5% of samples were positive for both PDGFR-α and β. The therapeutic effect of imatinib involves blocking the PDGFR receptor signalling pathway, which has been known to affect tumour growth directly, stabilise local vascularization and lymphangiogenesis, and promote tumour metastasis (Rosenberg and Mathew 2013). In human medicine, some studies emphasised the effect of metastatic suppression and elongation of median survival time in various tumour patients with distant metastasis who underwent TKI therapy (Massicotte et al. 2014; Saito et al. 2018). In addition, suppression of metastasis is a well-known characteristic of TKI. However, to the best of our knowledge, no veterinary case has been reported addressing the clinical application of imatinib to manage inoperable metastatic melanoma.

In the present case, the patient was classified as stage IV, since pulmonary metastasis was suspected, based on the staging scheme for dogs with oral melanoma (Bergman 2007). According to previous studies, the median survival time of WHO stage IV oral melanoma canine patients is 3 months (Kawabe et al. 2015). Initially, the patient was treated with immunotherapy, but after the detection of pulmonary nodules, imatinib therapy was initiated. After 3 months of imatinib therapy, the pulmonary lesions resolved, and the patient survived for 6 months following detection of pulmonary metastasis. This survival time is longer than the average survival time of 3 months reported for dogs with stage IV oral melanoma, indicating that the combined treatment with immunotherapy and imatinib therapy may have contributed to the extended survival observed in this case.

This report describes the presumably first study of the combination of immunotherapy and imatinib to treat incompletely resected oral malignant melanoma with suspected pulmonary metastasis in a dog. Although the suspected pulmonary metastatic lesions were not confirmed by cytologic or histopathologic examination, as well as post-mortem examination, regression of multiple nodules visible on regular thoracic radiography was evident. Therefore, the combination therapy of melanoma vaccination and imatinib could be considered a palliative therapeutic strategy in dogs with inoperable pulmonary metastatic oral melanoma without substantial adverse events.
